# Development of a Sensitive and Specific Polyclonal Antibody for Serological Detection of *Clavibacter michiganensis* subsp. *sepedonicus*

**DOI:** 10.1371/journal.pone.0169785

**Published:** 2017-01-09

**Authors:** Włodzimierz Przewodowski, Agnieszka Przewodowska

**Affiliations:** Plant Breeding and Acclimatization Institute – National Research Institute, Department of Potato Protection and Seed Science at Bonin, Poland; Agriculture and Agri-Food Canada, CANADA

## Abstract

The quarantine bacterium *Clavibacter michiganensis* subsp. *sepedonicus* (Cms) causes bacterial ring rot (BRR) in potato but is difficult to detect, hampering the diagnosis of this disease. ELISA immunoassays have not been widely used to detect Cms because commercially available anti-Cms antibodies detect mainly EPS-producing bacteria and can fail to detect strains that do not produce EPS. In the current study, we developed a new type of polyclonal antibody that specifically detects *Clavibacter michiganensis* subsp. *sepedonicus* bacteria irrespective of their EPS level. We first found that the presence of bacterial EPS precluded quantitative measurement of bacteria by currently available immunoenzymatic methods, but that washing Cms cells with acidic and basic buffers to remove EPS before analysis successfully standardized ELISA results. We used a mix of three strains of Cms with diverse EPS levels to generate antigen for production of antibodies recognizing Cms cells with and without an EPS layer (IgG-EPS and IgG-N-EPS, respectively). The resulting IgG-N-EPS recognized almost all Cms strains tested in this work regardless of their mucoidal level. The availability of this new antibody renders immunological diagnostics of Cms more sensitive and reliable, as our newly developed antibodies can be used in many type of immunoassays. This work represents an important step forward in efforts to diagnose and prevent the spread of BRR, and the methods and solutions developed in this work are covered by six Polish, one European and one US patents.

## Introduction

*Clavibacter michiganensis* subsp. *sepedonicus* (Cms) (Spickermann and Kotthoff 1914) Davis et al. 1984, which causes bacterial ring rot (BRR), is one of the most important pathogens of potato [1,2]. The genus *Clavibacter* consists of one species *Clavibacter michiganensis* (*Cm*), divided into five subspecies based on host specificity and biochemical and genetic characteristics. *Clavibacter michiganensis* subspecies are actinomycete plant pathogens residing mainly in the xylem vessels and inducing systemic symptoms, including wilting, stem cankers, and vascular discolouration [3]. While, *Cm*. subsp. *sepedonicus* (Cms) is responsible for BRR, *Cm*. subsp. *michiganensis* (Cmm) infects tomato, C*m*. subsp. *nebraskensis* (Cmn) induces wilt and blight in maize, *Cm*. subsp. *tessellarius* (Cmt) causes spots in wheat, and *Cm*. subsp. *insidiosus* (Cmi) causes wilting and stunting in alfalfa. The subspecies Cmm, Cms, and Cmi are subject to strict quarantine controls [4]. Virulence factors typical for *Clavibacter* include extracellular cellulases, hypersensitive response-inducing proteins, secreted enzymes and exopolysaccharides [4].

One of the most effective ways of reducing or eliminating BRR is through early detection of Cms, which is particularly important in the production, processing, and distribution of plant material. Hence, methods for BRR detection must be adequately sensitive and specific, as well as simple, fast, reliable, and reproducible. One barrier to detection is that Cms frequently occurs at low concentrations, resulting in an asymptomatic form of BRR known as latent infection. The presence of Cms at low concentrations can lead to latent spread of BRR in plants for several generations. Accordingly, the European and Mediterranean Plant Protection Organization (EPPO) Commission recommends the use of at least two different diagnostic tests based on different biological properties, including a pathogenicity test and appropriate physiological, biochemical, serological and/or molecular procedures [2].

The majority of Cms bacterial cultures on agar medium are of the mucoid colony type, although intermediate- and nonmucoid-type strains are also found [5,6]. Acidic bacterial exopolysaccharides (EPSs), which are produced in Cms, are also found in other subspecies of *C*. *michiganensis* [7]. Bacterial EPSs protect the bacterial cells against moisture loss, whereas they inhibit transpiration and cause wilting in the plant through the physical occlusion of vascular bundle walls [7]. EPSs consist of several (I–III) sugar moieties of similar chemical composition that are diverse in terms of the degree of agglomeration [8]. The occurrence of the IV moiety, consisting mainly of mannose, is characteristic only of Cms [9].

To date, assays for identifying Cms have been based on the analysis of their DNA [10–21], and fatty acid methyl ester [22] and protein profiles [23,24]. Much effort has focused on finding ways to identify Cms using serological methods. Earlier attempts involved immunodiffusion, latex agglutination, and indirect fluorescent antibody staining [25–27]. However, the sensitivity and specificity of these methods are unsatisfactory. Although Cms is a bacterial subspecies characterized by a relatively high phenotypic homogeneity [28], it exhibits diverse EPS levels, which makes immunological diagnosis difficult. Owing to numerous problems in the development of highly specific, sensitive polyclonal antibodies, most studies have been directed toward the production of monoclonal antibodies [29,26,27,30].

The use of monoclonal antibodies has significantly improved the specificity of immunofluorescence assay-type serological tests (IFAS); however, it has also led to reduced sensitivity of detection and increased costs. Moreover, 9AI monoclonal antibodies, which are used in IFAS directed against an antigen from the bacterial cell walls, are not suitable for enzyme linked immunosorbent assay (ELISA) [31]. By contrast, monoclonal antibodies directed against polysaccharide components are highly sensitive in ELISAs. However, such antibodies are useful only for strains with high EPS levels, and they produce false positive results due to cross-reactivity with *Clavibacter michiganensis* subsp. *michiganensis* and *Clavibacter michiganensis* subsp. *insidiosus* [31].

Although ELISA has been approved for diagnosis of Cms in North America [32], it has not yet been accepted by the EPPO as a reliable and functional detection method for Cms. Using monoclonal antibodies, this assay enables the detection of 1 × 10^5^ to 5 ×10^6^ bacterial cells/ml of suspension. With current polyclonal antibodies, Cms bacterial cells do not act as detectable antigens in ELISA. Instead, detection is mainly based on the free polysaccharides from mucus produced by Cms; thus, mucoid strains are detected more frequently, and those nonmucoid remain nearly undetected. All currently available polyclonal and monoclonal antibodies are characterized by high levels of nonspecific reactions giving false-positive results, which precludes a definitive diagnosis [28,33].

Mn-CS1 monoclonal antibodies directed against cell wall components and bacterial EPSs exhibit high sensitivity in double sandwich assay (DAS)-ELISA with extracellular polysaccharides, proteins from the cell walls, and the entire homologous antigen [30]. The possibility of using a combination of mono- and polyclonal antibodies in ELISA was previously investigated by Baer and Gudmestad [6]. All possible combinations of five polyclonal antibodies and one monoclonal antibody for identifying strains of Cms with diverse EPS levels were tested [6]. The highest sensitivity and specificity were obtained using a combination of a primary chicken polyclonal antibody as a capture and the mouse monoclonal antibody 1H3 as the sandwich. Using this system, the detection level for strains with high EPS levels was approximately 90% for cells in culture and 95% for cells from infected plant tissue. The intermediate ND9 strain was detected with 95% sensitivity. Unfortunately, the nonmucoid strains INM-1 and AK14 growing in culture medium were detected at only 36% and 24% sensitivity, respectively. In turn, the detection of nonmucoid strains from infected potato stems was 0–3%, regardless of the population density [6]. Another variant of serological testing was investigated by Smith et al. [34]. Bacteria were detected using a triple antibody sandwich (TAS-ELISA), in which the 1H3 monoclonal antibody constituted the primary antibody. However, the results were still unsatisfactory: nearly 20% of the samples giving positive PCR results were identified as negative in ELISA tests.

To promote detection efforts, the objective of the current study was to develop polyclonal rabbit anti-Cms IgG antibodies with immunological characteristics that would allow the detection of Cms via immunological methods, regardless of their EPS levels.

## Materials and Methods

### Bacterial isolates and growth conditions

The bacterial strains used in the study were obtained from the National Collection of Plant Pathogenic Bacteria (NCPPB, Central Science Laboratory, York, UK), the Belgian Coordinated Collections of Microorganisms (LMG/BCCM Rijksuniversiteit, Gent, Belgium), the Dutch Collection of Plant Pathogenic Bacteria (PD, Plant Protection Service, Wageningen, the Netherlands), the Collection of Plant Pathogens of the Plant Protection Institute (Poznań, Poland), and the Research Institute of Pomology and Floriculture (Skierniewice, Poland). Strains of *Clavibacter michiganensis* subsp. *sepedonicus* were cultured at 21°C using yeast glucose mineral medium (YGM). Among the 30 isolates of Cms investigated (Table 1), strains with high, intermediate, and low EPS levels were identified. Five- to seven-day-old cultures of bacterial cells derived from culture medium after double subculturing were used in this study. Strains of the other pathogens tested were cultured at ambient temperature in the appropriate culture media. All *C*. *michiganensis* subspecies were cultured on YGM medium, *Ralstonia solanacearum* on Yeast Peptone Glucose Agar (YPGA), *Pseudomonas* subspecies were cultivated on Nutrient Agar with 5% Saccharose (NAS) media, the same as *Agrobacterium fabrum*, *Erwinia amylovora*, and all *Pectobacterium* subspecies.

**Table 1:** Strains of *Clavibacter michiganensis* subsp. *sepedonicus*, *other C*. *michiganensis* subspecies, and diverse plant bacterial species analyzed in this study using commercially available kits and newly developed anti-Cms IgG.

Strain^a^	Origin	Host	Mucoid level	Year of isolation	Commercial kit			IgG anti-Cms against^e^		Detection by PCR^f^
					Loewe^b^	Agdia^c^	Adgen^d^	mucoid cells	nonmucoid cells	
*Clavibacter michiganensis* subsp. *sepedonicus*										
NCPPB 2136	United States	Potato	Fluidal	1945	**+**	**+**	**+**	**+**	**+**	**+**
NCPPB 2137	Canada	Potato	Intermediate	1968	**+**	**+**	**+**	**+**	**+**	**+**
NCPPB 2140	United States	Potato	Intermediate	1942	**+**	**+**	**+**	**+**	**+**	**+**
NCPPB 2913	Sweden	Potato	Fluidal	1977	**+**	**+**	**+**	**+**	**+**	**+**
NCPPB 3158	Italy	Potato	Intermediate	1981	**+**	**+**	**+**	**+**	**+**	**+**
NCPPB 3322	Belgium	Potato	Fluidal	1984	**+**	**+**	**+**	**+**	**+**	**+**
NCPPB 3323	Belgium	Potato	Intermediate	1984	**+**	**+**	**+**	**+**	**+**	**+**
NCPPB 3324	Belgium	Potato	Fluidal	1985	**-**	**+**	**+**	**+**	**+**	**+**
NCPPB 3326	Belgium	Potato	Fluidal	1984	**+**	**+**	**+**	**+**	**+**	**+**
NCPPB 3383	Norway	Potato	Rough	1977	**-**	**+**	**+**	**+**	**+**	**+**
NCPPB 3384	Norway	Potato	Fluidal	1983	**+**	**+**	**+**	**+**	**+**	**+**
NCPPB 3897	Ukraine	Potato	Rough	1994	**-**	**-**	**+**	**-**	**+**	**+**
NCPPB 3898	Ukraine	Potato	Rough	1994	**-**	**-**	**-**	**-**	**+**	**+**
NCPPB 4030	France	Potato	Fluidal	1977	**+**	**+**	**+**	**+**	**+**	**+**
NCPPB 4053	Sweden	Potato	Fluidal	1994	**+**	**+**	**+**	**+**	**+**	**+**
NCPPB 4216	Czech Republic	Potato	Intermediate	1997	**+**	**+**	**+**	**+**	**+**	**+**
NCPPB 4217	Czech Republic	Potato	Intermediate	1998	**+**	**+**	**+**	**+**	**+**	**+**
NCPPB 4218	Czech Republic	Potato	Intermediate	1999	**+**	**+**	**+**	**+**	**+**	**+**
NCPPB 4219	Czech Republic	Potato	Intermediate	1999	**+**	**+**	**+**	**+**	**+**	**+**
NCPPB 4220	Czech Republic	Potato	Fluidal	2000	**+**	**+**	**+**	**+**	**+**	**+**
NCPPB 4292	United States	Potato	Intermediate	2002	**+**	**+**	**+**	**+**	**+**	**+**
527	Poland	Potato	Rough	1994	**+**	**+**	**-**	**+**	**+**	**+**
529	Poland	Potato	Intermediate/rough	1993	**+**	**+**	**-**	**+**	**-**	**+**
758	Canada	Potato	Intermediate	1968	**+**	**+**	**+**	**+**	**+**	**+**
763	United States	Potato	Intermediate/rough	1942	**+**	**+**	**+**	**+**	**+**	**+**
LMG 5922	Argentina	Potato	Intermediate	1977	**+**	**+**	**+**	**+**	**+**	**+**
LMG 6382	Canada	Potato	Fluidal	1977	**+**	**+**	**+**	**+**	**+**	**+**
LMG 6385	Norway	Potato	Rough	1982	**-**	**+**	**+**	**-**	**+**	**+**
PD 406	Finland	Potato	Intermediate	1983	**+**	**+**	**+**	**+**	**+**	**+**
PD 680	Poland	Potato	Fluidal	1985	**+**	**+**	**+**	**+**	**+**	**+**
*Clavibacter michiganensis* subsp. *michiganensis*										
s1	Lithuania	Eggplant	Fluidal	ND	**+**	**-**	**+**	**+**	**+**	**-**
s2	Lithuania	Eggplant	Intermediate	ND	**+**	**-**	**+**	**+**	**+**	**-**
s7	Lithuania	Tomato	Intermediate	ND	**+**	**-**	**+**	**+**	**+**	**-**
s15	Lithuania	Tomato	Fluidal	ND	**+**	**-**	**+**	**+**	**+**	**-**
*Clavibacter michiganensis* subsp. *nebraskensis*										
NCPPB 2581	United States	Corn	Fluidal	1974	**-**	**-**	**+**	**+**	**+**	**-**
*Clavibacter michiganensis* subsp. *tessellarius*										
NCPPB 3664	United States	Winter wheat	Intermediate	1982	**-**	**-**	**+**	**+**	**+**	**-**
*Clavibacter michiganensis* subsp. *insidiosus*										
NCPPB 3032	ND	Alfalfa	Intermediate	1975	**+**	**-**	**+**	**+**	**+**	**-**
*Agrobacterium fabrum*										
C-58	Netherlands	Sweet cherry	Intermediate	ND	**-**	**-**	**+**	**-**	**-**	**-**
*Erwinia amylovora*										
659	Poland	Apple	Intermediate	1986	**-**	**-**	**+**	**+**	**-**	**-**
*Pectobacterium atrosepticum*										
676	Denmark	Potato	Intermediate	1952	**-**	**+**	**-**	**-**	**-**	**-**
9M	Poland	Calla Lily	Rough	2005	**-**	**-**	**+**	**+**	**-**	**-**
2M	Poland	Calla Lily	Rough	2005	**-**	**-**	**+**	**+**	**-**	**-**
1826	Poland	Calla Lily	Intermediate	2005	**-**	**+**	**+**	**+**	**-**	**-**
514	Poland	Potato	Intermediate	1976	**-**	**-**	**+**	**+**	**-**	**-**
NCPPB 549	United Kingdom	Potato	Intermediate	1958	**-**	**-**	**-**	**+**	**-**	**-**
*Pectobacterium carotovorum* subsp. *carotovorum*										
I-CS 2.1	Poland	Iris	Rough	2009	**+**	**-**	**+**	**+**	**-**	**-**
853	Poland	Calla Lily	Rough	ND	**+**	**-**	**+**	**+**	**-**	**-**
4M	Poland	Calla Lily	Intermediate	2005	**+**	**-**	**+**	**+**	**-**	**-**
*Pseudomonas fluorescens*										
837	Poland	Potato	Rough	1995	**-**	**+**	**-**	**+**	**-**	**-**
*Pseudomonas marginalis*										
7M	Poland	Calla Lily	Intermediate	2005	**-**	**-**	**-**	**-**	**-**	**-**
*Ralstonia solanacearum*										
NCPPB 4156	Netherlands	Potato	Fluidal	1995	**-**	**-**	**-**	**-**	**-**	**-**
PD 2762	Netherlands	Potato	Fluidal	1995	**-**	**-**	**-**	**-**	**-**	**-**
PD 2763	Netherlands	Potato	Fluidal	1995	**-**	**-**	**-**	**-**	**-**	**-**
PD 2764	Netherlands	Potato	Fluidal	1995	**-**	**-**	**+**	**-**	**-**	**-**
*Pseudomonas syringae* pv. *syringae*										
RIPF 110	Poland	Plum	Intermediate	2007	**-**	**-**	**+**	**-**	**-**	**-**
RIPF 760	Poland	Sour cherry	Intermediate	1999	**-**	**-**	**+**	**-**	**-**	**-**
PS 2905	Poland	Sour cherry	Intermediate	1978	**-**	**-**	**+**	**+**	**-**	**-**
*Pseudomonas syringae* pv. *tomato*										
Pst 3	Poland	Tomato	Intermediate	1999	**-**	**-**	**+**	**+**	**-**	**-**
*Pseudomonas veronii*										
6M	Poland	Calla Lily	Fluidal	2005	**-**	**-**	**+**	**+**	**-**	**-**
*Pseudomonas viridiflava*										
AWC—1	Poland	Chrysanthemum	Fluidal	ND	**-**	**-**	**+**	**+**	**-**	**-**

^a^Strains from the National Collection of Plant Pathogenic Bacteria (NCPPB, Central Science Laboratory, York, UK), the Belgian Coordinated Collections of Microorganisms (LMG/BCCM, Rijksuniversitet Gent, Belgium), the Dutch Collection of Plant Pathogenic Bacteria (PD, Plant Protection Service, Wageningen, the Netherlands), the Collection of Plant Pathogens of Plant Protection Institute (Poznań, Poland), and the Pomology Division of Research Institute of Horticulture (Skierniewice, Poland)

^b^Loewe kit containing polyclonal goat primary IgG against NCPPB 2140 and polyclonal goat secondary IgG conjugated with AP enzyme, DAS-ELISA method

^c^Agdia kit containing monoclonal mouse primary IgG and polyclonal rabbit secondary IgG conjugated with AP enzyme, DAS-ELISA method

^d^Adgen kit containing polyclonal primary IgG-anti Cms and polyclonal secondary IgG conjugated with AP enzyme, PTA-ELISA method

^e^Antibodies developed in this study

^f^PCR performed with PSA-1, PSA-R primers according to Pastrik [14], which is the only PCR procedure included in the Commission Directive 2006/56/EC on the control of potato ring rot [2]

+ = positive

- = negative

ND = not determined

**Fig 1:**
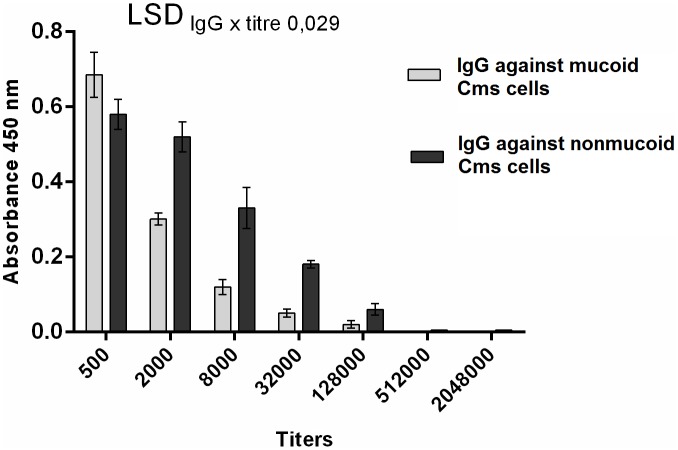
Representative calibration curves. These curves were determined for aqueous suspensions of Cms strains differentiated by mucous level (fluidal strain 758, intermediate strain 758 and rough strain NCPPB 4053). Measurement of absorbance of the bacterial suspensions was performed on EPOCH Microplate Spectrophotometer, Biotek at wavelength 630 nm.

For Cms, the number of colony forming units (CFU)/ml from bacterial cells in a suspension was determined based on a calibration curve plotted between the optical density of aqueous suspensions of each strain measured at 630 nm on a microplate ELISA reader and the number of colonies after 10-fold dilutions, followed by streak inoculations (100 μl) on solid YGM (Fig 1). For this bacterium, A_630_ = 0.1 corresponds to ~ 3 × 10^8^ CFU/ml. The number of CFU/ml of other bacteria tested in this work was determined by plating 10-fold dilutions of their aqueous suspension adjusted to A_630_ = 0,1 with water

### Preparation of Cms antigen for production of polyclonal antibodies in rabbit

**Fig 2:**
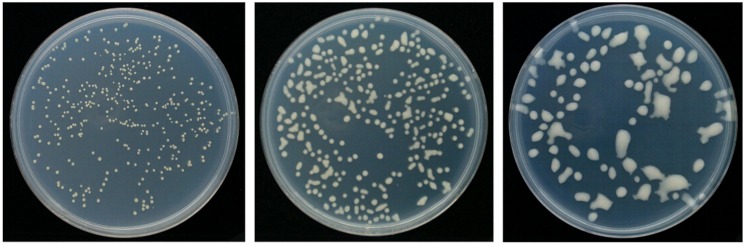
Colony morphology of the *Clavibacter michiganensis* subsp. *sepedonicus* strains selected for production of polyclonal antibodies in rabbit, according to their EPS levels. Left. Strain 527 –Rough colony type. Middle. Strain 758 –Intermediate colony type. Right. Strain NCPPB 4053 –Fluidal colony type.

To produce rabbit antibodies directed against bacteria producing EPS and bacteria without an EPS layer, a mix of three strains of Cms with diverse EPS levels was used. Strains with high EPS levels (NCPPB 4053), as well as intermediate (758) and low (527) EPS levels, were used (Fig 2). First, a10^8^ CFU/ml suspension was prepared for each strain, and the three strains were combined in a 1:1:1 ratio. The suspension was divided into two equal parts. The first part was used directly as an antigen to produce antibodies against bacteria producing EPS, and the second part was washed in acidic and basic buffers and used as an antigen to produce antibodies against EPS-free cells.

### Production of IgG against Cms cells with an EPS layer (IgG-EPS)

A suspension of three reference strains of Cms (527, 758, and NCPPB 4053) was centrifuged at 7,000 × g for 15 minutes, thermally inactivated at 80°C, and lyophilized. For subcutaneous immunization of rabbit, a 1% aqueous suspension of the lyophilisate with the addition of Gerbu adjuvant 100 (1:1, v/v) was used. The suspension (2 ml) was administered four times at 30-day intervals. On the first day of immunization, the complete adjuvant was administered, and in subsequent monthly intervals, incomplete adjuvant was administered.

Acquisition of blood from the rabbit and isolation of antibodies were performed according to [35]. Blood was collected from the marginal vein of the ear directly into the centrifuge tubes. After collection, the samples were incubated for 30 minutes at 37°C to form a clot and stored overnight at 4°C. The clot was gently separated from the walls of the tube and centrifuged at 1,000 × g at 4°C for 15 minutes. The serum was gently decanted from the precipitate, followed by the addition of NaN_3_ at a concentration of 0.02%, 1:10 dilution in H_2_O, and the addition of an equal volume of saturated ammonium sulfate. The solution was gently mixed and incubated for 60 minutes at room temperature to allow a precipitate to form. The mixture was then centrifuged for 5 minutes at 8,000 × g, the supernatant was removed, and the precipitate was dissolved in 0.5 × PBS buffer (pH 7.4) in a volume two-times higher than the initial volume of serum, followed by overnight dialysis with three changes of the same buffer containing 0.02% NaN_3_.

The dialyzed solution was applied to a column packed with DEAE cellulose equilibrated with 0.5 × PBS containing 0.02% NaN_3_, which was then used to elute the antibodies. After 3 ml fractions were collected, the absorbance ratio (A_280_/A_250_) of each fraction was measured. Fractions with A_280_/A_250_ ratios in the 2.5–2.7 range were combined and adjusted in 0.5 × PBS buffer to a concentration of 2 mg/ml, assuming the absorbance of 1 mg/ml IgG at λ = 280 nm is 1.4.

### Production of IgG against EPS-free Cms cells (IgG-N-EPS)

Suspensions of bacterial cells of the investigated Cms strains were washed three times with sterile ddH_2_O and centrifuged for 15 minutes at 7,000 × *g*. The supernatant was discarded and the remaining EPS on the centrifuged bacterial cells was washed twice for 5 minutes with 0.1 M glycine-HCl buffer, three times with sterile ddH_2_O, twice with 0.1 M glycine-NaOH buffer, and three times with sterile ddH_2_O, centrifuging the bacteria after each step for 15 minutes at 7,000 × g. EPS-free bacteria obtained using this procedure were lyophilized. Immunization of animals and isolation of antibodies were performed as described above for IgG directed against bacterial cells with an EPS layer. The procedure for washing off bacterial EPS is the subject of US and Polish patents [36,37].

This study was carried out in strict accordance with the recommendations in the Polish Legal ACT of 15 January 2015 “Protection of animals used for scientific or education purposes”. The protocol for was approved by the Local Committee on the Ethics of Animal Experiments in Szczecin (Permit Number: 10/2013, dated 02.07.2013). Immunizations and blood samples collections from animals were performed under local anesthetic lidocaine, and all efforts were made to minimize suffering.

### Trapped antibody-enzyme linked immunosorbent assay (PTA)-ELISA

After preparing bacterial suspensions in water at a concentration of 10^8^ CFU/ml, the bacteria were 1,000-fold diluted in coating buffer A (carbonate buffer, pH 9.6) to obtain a target concentration of 10^5^ CFU/ml. Then, 100 μl aliquots of the prepared suspensions of bacterial strains were loaded into microwells and incubated overnight at 4°C. The suspensions were washed three times with 1 × PBS-Tween20 (PBST) (pH 7.4) and covered with blocking buffer (5% Non fat dried milk powder (NFDM) in PBST, pH 7.4) using 200 μl per well, followed by incubation for 1 h at 37°C with shaking. The cells were washed three times with 1 × PBS-T (pH 7.4), followed by the application of newly developed anti-Cms rabbit IgG. Immunoglobulins were directed against bacterial cells with an EPS layer (IgG-EPS) and EPS-free bacteria (IgG-N-EPS), respectively, which were previously adjusted to a concentration of 0.1 mg/ml (for 1mg/ml IgG A_280_ = 1.4).

When evaluating the titer (expressed in a ratio that represents how many times the serum can be diluted until no reaction is found), the resulting IgG samples were diluted to various concentrations ranging from 1:500 to 1:2,048,000, whereas during evaluation of the specificity of the IgG with respect to the Cms strains and other bacterial pathogens tested, target dilutions of 1:2,000 (for anti-Cms IgG-EPS) and 1:8,000 (for anti-Cms IgG-N-EPS) were used. A 100 μl volume of the tested IgG solution was applied to each microwell and incubated for 3 h at 37°C with shaking. The solution was then washed five times with 1 × tris-buffered saline with 0,05% Tween 20 (TBST) pH 7.4 without NaN_3_, followed by the application of 5,000-fold diluted anti-rabbit IgG-Horseradish peroxidase (HRP) conjugate (Pierce) in conjugate buffer for HRP (1 × TBST with 2% PVP, 1% NFDM,) and incubation for 3 h at 37°C. The entire solution was washed five times with 1 × TBST, pH 7.4, combined with 100 μl of buffer containing substrate (200 μl 2 mg/ml tetramethylbenzidine (TMB) in DMSO + 9,8 ml 0.1 M sodium acetate, pH 6.0 + 300 μl 3% H_2_O_2_), and incubated for 20–30 minutes in the dark. The reaction was stopped by the addition of 15 μl 2M H_2_SO_4_ and the absorbance was measured at 450 nm. Serological tests using commercial DAS-ELISA kits purchased from Loewe (Cat. No.07064S/500), Agdia (Cms-Reagent Set—Cat. No. SRA 70002/1000) and PTA-ELISA kit Adgen (Cat. No. 1211-06/1000 NEOGEN Europe Ltd.) were also used according to the manufacturers’ instructions.

### Effect of EPS presence on immunodetection of Cms strains by DAS-ELISA

The effect of the presence of EPS produced by Cms cells on the sensitivity of DAS-ELISA results was evaluated using a commercially available kit from Loewe (Standard Double Antibody Sandwich Assay with polyclonal antibodies—DAS-ELISA), with three different mucoid strains of Cms, according to their EPS levels: 527, 758, and NCPPB 4053. Cms bacterial cells were prepared for testing in five different ways. The tested samples were bacterial cells suspended in water, cells washed with ddH_2_O, cells washed with acidic buffer (pH = 2.5), cells washed with basic buffer (pH = 10.5) and cells washed with two buffers (at pH = 2.5 and pH = 10.5) and than three times with ddH_2_O.

**Fig 3:**
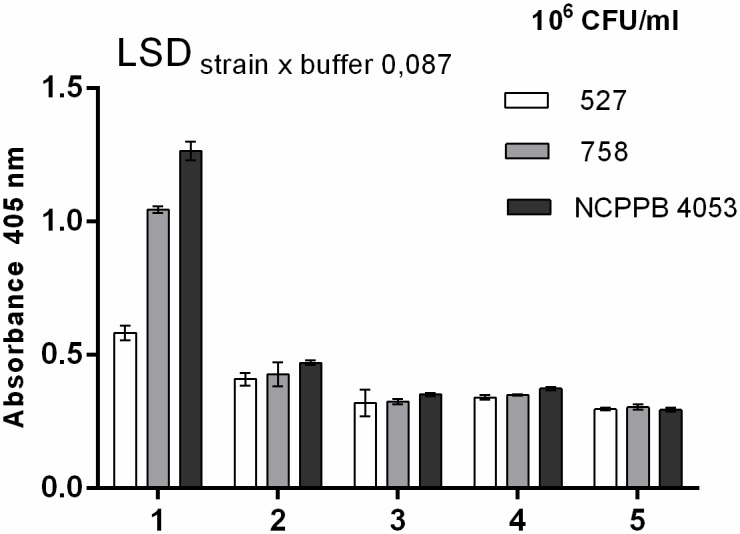
Detection of different Cms strains in terms of their EPS levels by DAS-ELISA (Loewe kit). Bacterial cells of three different strains (527 –Rough, 758 –Intermediate, NCPPB 4053 –Fluidal) were suspended: (1) in water, (2) washed three times with ddH_2_O, (3) washed twice at pH = 2.5 and three times with ddH_2_O, (4) washed twice at pH = 10.5 and three times with ddH_2_O, (5) washed twice at pH = 2.5, twice at pH = 10.5, and three times with ddH_2_O.Absorbance equal to at least two-fold the value of the blank sample was accepted as a positive result. All assays were performed twice in three replicates.

Absorbance equal to at least two-fold the value of the blank sample was accepted as a positive result. All assays were performed twice in three replicates, and the mean and standard deviation (SD) were calculated (Fig 3). The importance of the investigated factors on the analyzed characteristics was determined using two-way analysis of variance in Statistica 12 software. Student’s *t*-test was used to calculate the least significant difference (LSD) at a *P* = 0.001 significance level.

### Determination of the titer of newly developed anti-Cms immunoglobulins

**Fig 4:**
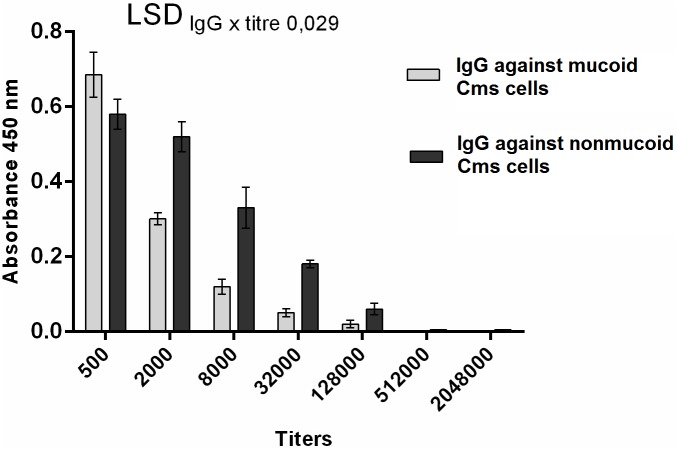
Detection of *Clavibacter michiganensis* subsp. *sepedonicus* mucoid and nonmucoid cells by PTA-ELISA with the newly developed antibodies. Assays were carried out with different titers of the obtained IgG against mucoid and nonmucoid cells. Immunization mixtures of strains NCPPB 4053, 758 and 527 were used at 10^5^ CFU/ml.

The titer of newly developed antibodies directed against Cms with EPS (IgG-EPS) and against Cms cells without EPS (IgG-N-EPS) was tested by PTA-ELISA using a series of 4-fold dilutions in the range of 500–2,048,000 (Fig 4). Antibodies were tested in the presence of two mixtures of Cms strains used earlier for immunization. The Student’s *t*-test was used to calculate the least significant difference (LSD) at a *P* = 0.001 significance level.

### Detection level of new IgG for three groups of Cms strains differentiated by mucoid level

Detection levels were tested via PTA-ELISA using the newly developed IgG against EPS-free bacteria (IgG-N-EPS) as primary antibody with starting concentration 0.1 mg/ml, diluted 1:2,000, and secondary antibody with horseradish peroxidase (HRP) diluted 1:5,000. Three groups of Cms strains were tested with the concentration of bacteria 10^5^ CFU/ml in all cases. Non-mucoidal strains of Cms (rough group) were represented by strains: 527, NCPPB 3898, LMG 6385, NCPPB 3897, NCPPB 3383. Strains with intermediate mucous level were NCPPB 2137, NCPPB 2140, 758, LMG 5922, NCPPB 3158 and those with a high level of mucus(fluidal strains) were NCPPB 4053, NCPPB 3324, LMG 6382, NCPPB 2913, NCPPB 3326.

### Field experiments

The possibility of using the newly developed antibodies to detect infections in the field was tested in samples of infected potato tubers. Potato tubers of the Gwiazda cultivar, which are sensitive to Cms bacterial infection, were used in a field experiment carried out in an experimental field belonging to the Plant Breeding and Acclimatization Institute in Poland. Six pathogenic Cms bacterial strains with different levels of bacterial EPS production were selected for the study. Strains 527 and LMG 6385 represented strains with low EPS level, strains 758 and NCPPB 3158 represented semi-EPS strains, and strains NCPPB 2913 and NCPPB 3326 represented strains producing large amounts of EPS. Healthy potato tubers were used as the negative control.

Each bacterial cell suspension of each strain inoculum was prepared at a concentration 2.5 × 10^8^ CFU/ml in PB. After harvest, the tubers from each plant were observed. The number of tubers with clear symptoms of the disease and rotten tubers was noted, and the rotten tubers were not further analyzed. PCR and ELISA tests were performed on the remaining tubers to evaluate asymptomatic infection and infection of the tubers with bacteria causing potato ring rot (PRR) disease. Samples of potato tubers infected with Cms consisted of approximately 1 g slices obtained from the filial tubers of all five plants per replicate. The PCR and ELISA tests were performed according to the methodology detailed in the Commission Directive 2006/56/EC [2]. The experiment was performed with three replicates. The importance of the effects of investigated factors on the analyzed characteristics was determined by univariate analysis of variance at a significance level of 0.0001 with Statistica 12 software.

## Results

### Increase in sensitivity of DAS-ELISA as an effect of the presence of bacterial EPS

We aimed to produce antibodies to detect Cms cells irrespective of their EPS levels and, if possible, without compromising the sensitivity of the assay. We began by evaluating the effect of EPS on ELISA sensitivity using from the commercial Loewe kit (Fig 3). We found that bacterial EPS components were the main antigens reacting with with IgG in the kit. Even though we used almost identical concentrations of each Cms strain (10^6^ CFU/ml) in the tests, a high absorbance level was detected for the highly mucoid strain 4053, a moderate level was detected for strain 758, and the lowest level was detected for strain 527, which produced the least EPS (Fig 2). After the removal of EPS from the bacteria by triple washing with ddH_2_O, the DAS-ELISA results were better correlated with the (equal) amounts of bacterial cells in the suspension. The removal of EPS with acid and alkaline buffer completely standardized the results of DAS-ELISA, although it also reduced the sensitivity of the assay. There was a significant difference (two-way analysis of variance at a significance level of *P* = 0.05 with the Student’s t-test) in absorbance levels between samples washed with water and those washed with acidic and alkaline pH buffers. Since the production of high-quality antigen requires mucus to be washed from Cms cells in the most effective manner, we selected the acidic/alkaline method as the most promising method investigated.

### Comparison of antibody titers and immunocharacteristics

To precisely determine the influence of the presence of the EPS layer on the detection of Cms using our newly developed antibodies, we evaluated the antibody titer with respect to bacterial cells in aqueous suspensions in which EPSs were removed by three washes in ddH_2_O and in suspensions of EPS-free bacteria that were washed with low and high pH buffers (method chosen based on the results described above). We evaluated the titers of the obtained antibodies by PTA-ELISA using immunization mixtures of strains 4053 NCPPB, 758, and 527 at a concentration of 10^5^ CFU/ml. As expected, antibodies directed against EPS-producing bacterial cells exhibited the highest affinity to bacteria with an EPS layer in aqueous suspension and a lower affinity to partially and completely EPS-free cells (Table 2). Conversely, antibodies directed against the EPS-free bacterial cells reacted preferably with EPS-free bacteria and weakly with partially EPS-free cells washed with ddH_2_O and with bacteria surrounded by an EPS layer in an aqueous suspension. Additionally, antibodies directed against EPS-free bacterial cells were less sensitive to variable amounts of EPS layer in the samples. The detection of EPS-producing cells in aqueous suspension was maintained at the same level as that of bacteria for which EPS were washed off with water and low pH buffer. The titer for these samples was 1:32,000. For both antibodies tested, the highest titer achieved was 1:128,000.

**Table 2:** Properties of the newly developed antibodies against Cms bacterial cells (strains NCPPB 4053, 758, 527) using mucoid and nonmucoid cells. Titers required for detection were estimated in each of the cases by the PTA-ELISA method using 10^5^ CFU/ml of aqueous suspensions of bacteria and suspensions for which EPS components were gradually washed off.

IgG	Cms cell suspension treatment	IgG dilution							Blank(no IgG)
		1:500	1:2,000	1:8,000	1:32,000	1:128,000	1:512,000	1:2,048,000	
**IgG against mucoid Cms cells**	Suspension in water	+	+	+	+	+	-	-	-
	Washing three times with sterile water	+	+	+	+	-	-	-	-
	Washing at pH 2.5	+	+	-	-	-	-	-	-
	Washing at pH 10.5	+	+	+	-	-	-	-	-
	Washing at pH 2.5 and 10.5	+	+	-	-	-	-	-	-
**IgG against nonmucoid Cms cells**	Suspension in water	+	+	+	+	-	-	-	-
	Washing three times with sterile water.	+	+	+	+	-	-	-	-
	Washing at pH 2.5	+	+	+	+	+	-	-	-
	Washing at pH 10.5	+	+	+	+	-	-	-	-
	Washing at pH 2.5 and 10.5	+	+	+	+	+	-	-	-

+ = positive result from PTA- (at least two-fold higher compared to negative control)

− = negative result from PTA-ELISA (less than two-fold higher compared to negative control)

The antibodies were then tested against two mixtures of Cms used earlier for immunization (Fig 4). At low dilution (500×) of antibody, both mixtures were detected at a similar level. At higher dilutions, IgG-EPS antibodies were less sensitive towards bacterial cells producing EPS than IgG-N-EPS antibodies. Finally, the titers of both tested antibodies were determined at a level of 128,000. Significant differences between the two tested antibodies were evident in every titer in the 500–128,000 range (Fig 4).

We next investigated the sensitivity of three different commercially available diagnostic kits, as well as of the antibodies developed in our laboratory, in the presence of two extremely diverse Cms strains with respect to the amount of the secreted mucus (nonmucoid 527 and fluidal mucoid NCPPB 4053 strain) (Table 3). The sensitivity of detection of the nonmucoid strain 527 and the fluidal mucoid NCPPB 4053 strain depended, to a large extent, on the EPS level in the strain tested (Table 3). The Loewe kit detected the nonmucoid strain 527 at an estimated concentration of 10^6^ CFU/ml bacterial cells. The sensitivity of this kit for the identification of the fluidal mucoid NCPPB 4053 strain was the best among the diagnostic kits tested. Positive results for this fluidal mucoid strain were obtained at 10^3^−10^4^ CFU/ml. The trend toward better detection of mucoid strains was also observed using the Agdia and Adgen kits, and the antibodies developed in our laboratory directed against Cms cells producing EPS.

**Table 3:** Comparison of the sensitivity of different ELISA tests for detecting Cms bacterial cells. Strains tested were 527 and NCPPB 4053.

Bacterial suspension (CFU/ml)	Loewe^a^		Agdia^b^		Adgen^c^		Newly developed IgG			
							Against mucoid Cms cells		Against nonmucoid Cms cells	
	527	NCPPB 4053	527	NCPPB 4053	527	NCPPB 4053	527	NCPPB 4053	527	NCPPB 4053
10^7^	+	+	+	+	-	+	+	+	+	+
10^6^	+	+	-	+	-	+	(+)	+	+	+
10^5^	-	+	-	+	-	+	-	+	+	+
10^4^	-	+	-	(+)	-	-	-	+	+	(+)
10^3^	-	(+)	-	-	-	-	-	-	-	-
10^2^	-	-	-	-	-	-	-	-	-	-
NC1	-	-	-	-	-	-	-	-	-	-
NC2	-	-	-	-	-	-	-	-	-	-

+ = positive result from ELISA (more than two-fold higher than the negative control)

(+) = weak positive (approximately two-fold higher than the negative control)

- = negative result from ELISA (less than two-fold higher than the negative control)

NC1—Negative control 1—Coating buffer

NC2—Negative control 2—Potato extract diluted 1:100 in coating buffer

^a^Loewe kit containing polyclonal goat primary IgG against the Cms strain NCPPB 2140 and polyclonal goat secondary IgG conjugated with AP enzyme, DAS-ELISA method

^b^Agdia kit containing monoclonal mouse primary IgG and polyclonal rabbit secondary IgG conjugated with alkaline phosphatase AP enzyme, DAS-ELISA method

^c^Adgen kit containing polyclonal primary IgG-anti Cms and polyclonal secondary IgG conjugated with AP enzyme, PTA-ELISA method

**Fig 5:**
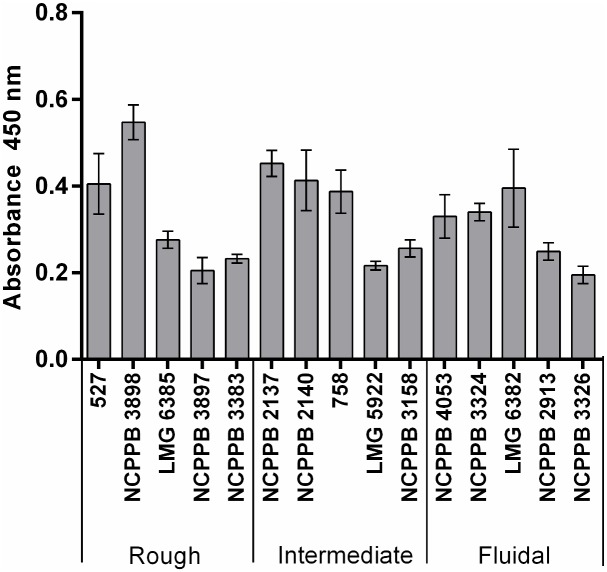
Determination of *Clavibacter michiganensis* subsp. *sepedonicus* levels using newly developed polyclonal antibodies against mucus-free bacterial cells tested by PTA-ELISA. Primary antibodies at 0.1 mg/ml were diluted 1:2,000, and secondary antibodies with horseradish peroxidase (HRP) were diluted 1:5,000. The concentration of bacteria was 10^5^ CFU/ml in all cases. Three groups of Cms strains were tested: 1) rough group (527, NCPPB 3898, LMG 6385, NCPPB 3897, NCPPB 3383), intermediate (NCPPB 2137, NCPPB 2140, 758, LMG 5922, NCPPB 3158) and fluidal strains (NCPPB 4053, NCPPB 3324, LMG 6382, NCPPB 2913, NCPPB 3326). All assays were performed twice in three replicates.

The sensitivity of the Agdia kit for the nonmucoid strain was quite low (about 10^7^ CFU/ml), whereas for the fluidal mucoid strain, the sensitivity was estimated to be 10^4^−10^5^ CFU/ml. With respect to Adgen kit, strain 527 could not be detected at any tested concentration, and the sensitivity for the mucoid strain was about 10^5^ CFU/ml. The antibodies produced in this work and directed against mucoid Cms cells were more efficient in detection of the mucoid strain (sensitivity about 10^4^ CFU/ml) than in detection of the nonmucoid strain (sensitivity about 10^6^–10^7^ CFU/ml), whereas those produced against nonmucoid Cms cells were characterized by high sensitivity (about 10^4^ CFU/ml) for Cms cells, regardless of their mucoid level (Table 3). We evaluated the detection level for the three groups of Cms in terms of mucoid level via PTA-ELISA using antibodies directed against nonmucoid cells as primary antibodies and conjugates of secondary antibodies with HRP (Pierce) (Fig 5). There was no clear correlation between the EPS levels of the strains and the absorbance level. In each group of bacteria, we observed strains exhibiting a relatively high response in the assay, as well as strains for which the signal strength was considerably lower. Only one strain (NCPPB 3326) exhibited a signal lower than A_450_ = 0.2.

### Antibody specificity

We investigated the specificity of various available antibodies by PTA-ELISA using commercially available ELISA assay kits from Adgen, Agdia, and Loewe and our newly developed antibodies (Table 1). The identity of all Cms strains examined was initially confirmed by RT-PCR using primers developed by Pastrick [16]. The assays were performed using 30 strains of *Clavibacter michiganensis* subsp. *sepedonicus*. The detection levels for particular Cms strains differed depending on the diagnostic kit utilized. The DAS-ELISA kit from Loewe gave a negative result for five out of 30 Cms strains. Four of these strains were nonmucoid strains and one (NCPPB 3324) was characterized by a high mucoid level. The PTA-ELISA diagnostic kit from Adgen (containing polyclonal IgG) gave a negative result for three of the Cms strains (NCPPB 3898 and 527, with low mucoid levels, and the intermediate/rough strain 529). Similar results were obtained using a DAS-ELISA kit with mono- and polyclonal antibodies purchased from Agdia, for which the nonmucoid strains NCPPB 3897 and NCPPB 3898 produced negative results in ELISA. Polyclonal rabbit antibodies produced in our laboratory directed against bacteria surrounded by EPS gave a negative result for three Cms strains, including two (NCPPB 3897 and NCPPB 3898) not detected by the Agdia kit and one (LMG 6385) not detected by the Loewe kit.

The newly developed antibodies directed against nonmucoid bacteria provided the best specificity of detection of the Cms bacterial strains examined. Only one intermediate/rough strain (529) was not detected using these antibodies. This strain was also not detected using the Adgen diagnostic kit. We previously found that this strain was not recognized as pathogenic in a biotest on eggplant (not shown). The diagnostic kits from Adgen and Loewe, like our newly developed IgG-EPS, also cross-reacted with all bacterial strains of *Clavibacter michiganensis* subsp. *michiganensis*. Only the Agdia kit gave a negative result for this pathogen.

We detected nonspecific reactions toward potato pathogens using the Adgen kit, which detected four out of the six strains of *Pectobacterium carotovorum* subsp. *atrosepticum* tested, including one isolated from potato plants. The Agdia kit gave a positive result for two strains, whereas antibodies developed in our laboratory directed against cells with EPS detected five out of six tested strains of this pathogen. IgG-EPS, like the Loewe and Adgen kits, nonspecifically detected all *Pectobacterium carotovorum* subsp. *carotovorum* strains. By contrast, the Agdia kit and antibodies directed against nonmucoid cells did not react with any strain of this pathogen.

We obtained a positive result for an *Erwinia amylovora* strain using the Adgen kit and the newly developed antibodies directed against EPS-producing cells. *Pseudomonas fluorescens* was also nonspecifically detected by the antibodies and Adgen kit, which also detected one of the *Ralstonia solanacearum* strains. Gram-positive bacterial strains *Bacillus subtilis* NCPPB 3272 and *Streptomyces scabies* NCPPB 2537 were not detected by any of the commercial kits or the new antibodies IgG-EPS and IgG-N-EPS.

### Field samples

We investigated the utility of the newly developed antibodies for identifying infections caused by Cms bacteria in potato plants using tubers of the Gwiazda cultivar, which is characterized by high sensitivity to infection with Cms bacteria. We selected six pathogenic strains with different EPS levels, including non-mucoid, semi-mucoid, and highly mucoid, with two strains per group (Table 4).

**Table 4:** Detection of *Clavibacter michiganensis* subsp. *sepedonicus* with newly developed IgG directed against cells from this bacterial species without the exopolysaccharide layer (IgG-N-EPS) in the field.

	Rough Cms strains		Intermediate Cms strains		FluidalCms strains		Negative control
	527	LMG 6385	758	NCPPB 3158	NCPPB 2913	NCPPB 3326	
**Pathogenity**^a^	**	*	*	*	**	***	-
**Absorbance (405 nm)**	0.360	0.158	0.250	0.182	0.280	0.350	0.045
**SD**	±0.009	±0.009	±0.005	±0.007	±0.01	±0.008	± 0.004
**PCR**^b^	+	+	+	+	+	+	-

Least significant difference (LSD) = 0.018 was determined using univariate analysis of variance at a significance level of 0.0001

Negative control—Non-inoculated

^a^Pathogenity of Cms strains determined by bioassay according to Directive 2006/56/EC [2].

^b^PCR performed with PSA-1, PSA-R primers according to Pastrik [16], which is the only PCR procedure included in the Commission Directive 2006/56/EC on the control of potato ring rot [2].

*weakly pathogenic,

** pathogenic,

*** strongly pathogenic

+ = positive result from ELISA (at least two-fold higher compared to negative control)

- = negative result from ELISA (less than two-fold higher compared to negative control)

We tested the selected tubers from infected plants using PCR and PTA-ELISA. In the PTA-ELISA test, samples with at least twice the absorbance value of the negative control, which consisted of extract from non-inoculated potato tubers, were considered to be positive. The PCR results confirmed the presence of the pathogen in all samples investigated. Therefore, the newly developed antibodies revealed latent infection in all samples. The absorbance values obtained in the PTA-ELISA test varied depending on the strain, with values ranging from A_405_ = 0.158 (± 0.009) for the LMG 6385 rough strain to A_405_ = 0.360 for the 527 strain.

Further analysis of the results indicated that absorbance levels were higher for strains producing the largest amounts of EPS (NCPPB 2913 and NCPPB 3326), which are characterized by high pathogenicity. Interestingly, among the rough strains, LMG strain 6385, which is weakly pathogenic and produces small amounts of EPS, achieved an absorbance level of 0.158 in the PTA-ELISA test. Strain 527, which is characterized by low amounts of EPS but medium pathogenicity, produced an absorbance level of 0.360, which is even greater than that of the highly mucoid, pathogenic NCPPB 3326 strain (Table 4). Statistically significant differences (*P* = 0.0001) were observed among strains regardless of mucoidal group. The difference in absorbance between only two strains, 527 (rough) and NCPPB 3326, was not significantly different (LSD = 0.018).

## Discussion

*Clavibacter michiganensis* subsp. *sepedonicus* is considered a quarantine bacterium in the European Union [38] that is particularly difficult to detect and diagnose. Numerous problems that contribute to the poor detection of this pathogen in potato tissues have been reported. One problem is the frequent occurrence of Cms at low concentrations, resulting in an asymptomatic form of the disease in plants [39,40]. While the currently used Cms detection methods have been verified, they do have some limitations. The use of just one method does not often give the correct diagnosis with 100% certainty. Therefore, in accordance with the accepted phytosanitary requirements, at least two assays based on different biological properties must be used, along with a pathogenicity assay [2],

Due to the morphological diversity of strains of this bacterium, the detection of Cms using serological methods poses a problem for the diagnosis of the pathogen responsible for BRR [41]. The indirect immunofluorescence (IF) test is the most commonly used serological method and is recommended by the EPPO for the detection of Cms [42,43]. However, although popular in North America, the use of ELISA for the identification of Cms [44–46] has not been approved as a screening test by the EPPO. The differences in absorbance levels in ELISA may stem from the diverse ability of individual Cms bacterial strains to colonize tissues. Indeed, low mucoid strains at the initial stage of cultivation usually have lower population levels and prompt weaker reactions in ELISA tests than semi-mucoid and highly mucoid strains [6]. However, some strains with initially low absorbance levels exhibit considerably increased population values over time. For example, after 103 days of cultivation, the Cms INM-1 strain achieved a 2.5-fold higher number of bacterial cells than the semi-mucoid ND9 strain, but their absorbance in the ELISA test was almost five-fold higher [6].

Monoclonal antibody-based immunological diagnosis appears to be more reliable than ELISA for identifying Cms strains with diverse EPS levels [31,30]. The use of monoclonal antibodies in the IF test provides high specificity [31], with the detection level of Cms reaching 10^4^ bacterial cells in 1 ml of potato tissue extract [6]. Inexpensive, less specific polyclonal antibodies are sometimes used to increase the sensitivity of serological methods. However, this method frequently produces false positive results due to cross-reactivity with other bacteria [47] and cannot be used to detect Cms strains without EPS layers [6,48].

Problems with the use of antibodies for Cms detection occur because Cms bacterial cells are not the only detectable antigens: soluble EPS produced by these cells, the amounts of which may differ depending on the number of cells, also function as antigens. Both IF and ELISA allow Cms to be detected in potato tubers and stems; while the immunofluorescence method exhibits greater sensitivity and specificity for potato tubers, the ELISA provides higher sensitivity toward the pathogen in potato stems [45].

In the present study, we demonstrated that the presence of bacterial EPS precludes quantitative measurement of bacteria using immunoenzymatic methods. The sensitivity of ELISA depends primarily on the amount of EPS produced by a particular strain of Cms and not on the actual number of cells in suspension. Washing of Cms bacterial cells with acid (pH 2.5) or alkaline (pH 10.5) buffer removes most EPS components and allows the ELISA results to be standardized. This effect is not obtained with even repeated washing of bacterial suspensions with water. Developing an effective method for EPS removal from bacterial cells by washing them in low and high pH buffer enabled significantly better serological evaluation of the number of bacterial cells, regardless of the EPS level, in the Cms strains examined.

Efficient washing of EPS from the bacterial cells allows ELISA results to be standardized and to be considered semiquantitative. However, the use of this additional step prior to the ELISA test is unenforceable in investigations of plant health. In the present study, we used bacterial cells that were washed to remove EPS to prepare the antigen for animal immunization. Subsequently, we developed a new type of antibody directed against Cms. Both whole Cms cells with an EPS layer and EPS-free bacterial cells were used as antigens. Among the antibodies, we obtained IgG-N-EPS directed against nonmucoid bacterial cells, which is characterized by high affinity toward the tested Cms strains and weak interactions with other bacteria. Moreover, the newly developed antibodies did not react with any other potato pathogen examined.

The interaction between the new antibodies directed against EPS-free bacteria and certain subspecies of *Clavibacter michiganensis* does not affect the proper diagnosis of Cms due to the absence of these pathogens in potato tissue. Nonspecific interactions, particularly cross-reactions between these pathogens, were previously observed by De Boer et al. [25,27]. Antibodies directed against bacteria producing EPS detected all Cms strains with high sensitivity; however, they were also characterized by nonspecific interactions with other potato pathogen, such as *Pectobacterium atrosepticum*, making them ineffective for serological diagnosis. According to Miller [49] and Kokoskowa and Pankova [50,51], *Pseudomonas fluorescens* is the most cross-reacting bacterium in serological assays directed against Cms. In the current study, positive results for this pathogen were obtained using an Agdia kit and antibodies against bacteria with EPS. There were no positive results for this pathogen using other tested commercial kits and our new antibodies directed to cells without EPS.

We found that none of the commercially available kits (Loewe, Agdia, and Adgen) detected all of the investigated Cms strains. The Agdia kit with monoclonal antibodies and the Adgen kit for PTA-ELISA failed to detect some of the investigated Cms strains. Moreover, the Loewe kit based on polyclonal antibodies did not detect all tested Cms strains. The Loewe kit, unlike the Adgen kit, was characterized by fewer nonspecific interactions with other pathogens.

Our results confirm that the commercially available anti-Cms antibodies mainly react with components of bacterial EPS, but they react poorly with bacterial cells. The diverse EPS levels of certain Cms strains preclude the measurement of bacterial cells by immunoenzymatic methods. Rabbit antibodies produced by this method and directed against EPS-free Cms cells do not react with EPS components, enabling better evaluation of Cms, regardless of the EPS levels of the strains, and they do not exhibit nonspecific reactions with other potato pathogens. Some nonspecific reactions of IgG-N-EPS were observed for other *Clavibacter michiganensis* subspecies, but none of these were potato-specific pathogens (*Clavibacter michiganensis* subsp. *michiganensis*, *Clavibacter michiganensis* subsp. *nebraskensis*, *Clavibacter michiganensis* subsp. *tessellarius*, *Clavibacter michiganensis* subsp. *insidiosus*).

The development of new polyclonal antibodies for Cms detection with relatively high specificity represents a new avenue for the development of a serological method to detect extremely diverse bacteria in terms of mucoid level. Among the commercially available kits that use both mono- and polyclonal anti-Cms antibodies, none detected the investigated Cms strains at a rate of 100%. The best results were obtained using the newly developed polyclonal antibodies IgG-N-EPS. These antibodies failed to detect only one of the Cms strains examined (529), whereas all other Cms strains were detected regardless of their EPS level. The currently used ELISA immunoassays make it possible to test a large number of samples simultaneously; however, they do not allow for the morphological assessment of bacterial cells. In turn, IFAS allows for morphological detection of bacterial cells, but substantially fewer detection tests can be performed simultaneously. Therefore, there is a need for novel, rapid, sensitive, specific immunodiagnostic assays that do not exhibit such limitations [52]. The current methods for Cms isolation recommended by the EPPO generally require mechanical separation of bacterial cells from the material (centrifugation, decantation, and so on) and do not allow the components present in the tested samples to be fully discarded, adversely affecting the results of diagnostic assays.

Components such as starch grains and plant tissue fragments cover bacterial cells when viewed under a microscope during IFAS detection of cells. Moreover, the presence of bacterial EPS and plant components in immunoassays may induce high backgrounds, which interferes with the reaction. Molecular assays are characterized not only by high specificity, but also by sensitivity to inhibitors that hinder amplification of the genetic material, giving false-negative results.

Isolating bacteria from environmental samples and producing pure cultures for subsequent assays are difficult due to the lack of selective media for Cms. Other fast-growing microorganisms that inhibit and prevent the growth of Cms often contaminate the resulting preparations. These problems might be solved by applying the preliminary stage of immunoconcentration of Cms bacterial cells from the tested samples using specific antibodies. During this process, it is extremely important to use an antibody immunoconcentration stage that can selectively capture Cms cells regardless of their EPS level.

The antibodies developed in the current study are sensitive and specific. Therefore, they provide the opportunity to develop immunosorbents that allow for efficient isolation of Cms characterized by different mucoid contents while simultaneously removing the undesirable components and other microorganisms that may hinder diagnosis.
